# Iron folic acid supplementation adherence level and its associated factors among pregnant women in Ethiopia: a multilevel complex data analysis of 2019 Ethiopian mini demographic and health survey data

**DOI:** 10.3389/fnut.2024.1348275

**Published:** 2024-02-16

**Authors:** Habtamu Temesgen, Wubetu Woyraw, Fentaw Wassie Feleke, Getachew Sale Mezgebu, Kefyalew Taye, Tadesse Awoke

**Affiliations:** ^1^Department of Nutrition, College of Health Science, Debre Markos University, Debre Marqos, Ethiopia; ^2^School of Human Nutrition, College of Agriculture, Hawassa University, Hawassa, Ethiopia; ^3^Department of Public Health, College of Health Science, Woldia University, Woldia, Ethiopia; ^4^Department of Public Health, College of Health Science, Ambo University, Ambo, Ethiopia; ^5^Institute of Public Health, College of Health Science, University of Gondar, Gondar, Ethiopia

**Keywords:** non-adherence, iron folic acid supplementation, Mini-EDHS 2019, women, Ethiopia

## Abstract

**Background:**

Iron and folic acid deficiency is a worldwide public health concern, particularly in low and middle-income countries. In Ethiopia, adherence to iron and folic acid supplements is still very low. Despite the fact that a number of studies on IFA supplementation have been conducted in Ethiopia, they do not indicate a nationwide problem and do not use advanced models to demonstrate clustering effects. The purpose of this study was to assess the level of non-adherence to iron folic acid supplementation and predictors among pregnant women in Ethiopia.

**Objective:**

To assess iron folic acid supplementation adherence level and its associated factors among pregnant women in Ethiopia using data from the 2019 Mini-Ethiopian demographic health survey.

**Methods:**

The Mini Ethiopian Demographic and Health Survey 2019 data were obtained from the official database website of the Demographic and Health Survey program (http://dhsprogram.com). The analysis included a sample of 2,356 weighted study participants. A multivariable multilevel mixed-effects logistic regression model was used Variables with *p*-values less than 5%) was reported as statistically significant variables in the multivariable analysis.

**Results:**

The proportion of mothers who did not adhere to iron and folic acid supplements was 81.03% (95 %CI, 79.39, and 82.56). Birth interval less than 2 years [AOR: 2.03; 95% CI: 1.12, 3.66], women ever born less than six children [AOR: 1.99; 95% CI: 1.09, 3.64], starting ANC visit during first trimester [AOR: 2.74; 95 %CI: 1.03, 7.30], region [AOR = 0.24; 95% CI: 0.10], and having a high no ANC visit in the community [AOR = 1.77; 95% CI: 1.08, 2.88] were statistically significant factors. There was Intra-Custer Correlation (ICC = 17.72%), indicating that 17.72% variability in non-adherence levels was due to clusters.

**Conclusion and recommendation:**

In Ethiopia, nearly four out of every five pregnant women did not receive iron folic acid supplementation for the recommended periods. Birth intervals, number of children, timing of ANC visits, region, and community level no ANC service were significant factors for non-adherence IFAS. As a result, the community, govern metal and non-governmental sectors enacting on health should focus on reducing non-adherence through tailored interventions on factors that influence it.

## Introduction

Iron and folic acid deficiency is a global public health concern, particularly in low and middle-income countries (1). Iron with folic acid is an important micronutrient for physiological function, growth, and development, as well as the mother’s and her fetus’s survival during pregnancy and later life. Similar to other nutrients, the demand for and scarcity of iron and folic acid increase during pregnancy to meet the daily requirement for the fetus’s life development and growth (2).

The World Health Organization (WHO) estimates that iron deficiency is responsible for approximately 50% of all anemia cases (3). More than 56 million pregnant women suffer from anemia worldwide, with Africa accounting for two-thirds (17.2 million) (4) According to the Ethiopian Demographic and Health Survey (EDHS), the prevalence of anemia among women aged 15–49 years fell from 27 to 17% in 2011, but increased to 24% in 2016, with pregnant women accounting for 29% (5). Similarly, different studies conducted in Ethiopia show that the prevalence of anemia among pregnant women was between 21 and 54% (6–10).

A large population based study conducted among 22 Sub-Saharan African countries showed that only 28.7% of women took iron supplements for ≥90 days during pregnancy (11). The Ethiopian demographic health survey of 2016 reported that only 5% of pregnant women took an iron with a folic acid tablet for 90 days and 58% of pregnant women did not at all during their time of pregnancy (12). A meta-analysis studies done in 2019 and 2020 show that the pooled estimated prevalence of adherence to iron-folic acid supplementation among pregnant women in Ethiopia was 41.38 and 46.15% ranging from the lowest 17.1% to the highest 74.5%, respectively, (13, 14).

Findings from different literature shows that poor knowledge and awareness about anemia during pregnancy, inaccessibility of IFAS, fear of side effects, believed to be responsible for not conforming to the recommended IFAS during pregnancy, utilization of ANC services, inadequate supply of IFA tablets, poor counseling and lack of knowledge on anemia were the identified associated factors for poor adherence to IFAS (15–17).

Iron and folic acid supplementation (IFAS) with optimal adherence is the main cost-effective strategy for prevention and control of iron and folate deficiency anemia during pregnancy (18). IFAS during pregnancy was shown to reduce the risk of all types of maternal anemia by 70% and iron deficiency anemia by 57% (19).

World Health Organization recommends that all pregnant women receive a standard dose of 30–60 mg iron and 400-μg (0.4 mg) folic acid beginning as early as possible during gestation. Ideally, women should take 180 tablets before delivery; however, many countries, including, Ethiopia aim for women to receive 90+ tablets during pregnancy (20).

Reducing anemia is an important component of achieving women’s and children’s health. The second global nutrition targeted a 50% reduction of anemia in women of reproductive age in 2025 (21). Similarly, Ethiopia National Nutrition Program (NNP II) also set a key target to increase the number of women receiving iron-folic acid supplements for more than 90 days during pregnancy to 40% by 2020 (22).

Despite the fact that various studies have been conducted in Ethiopia to assess the magnitude and factors associated with iron supplementation adherence, these studies were conducted on a small number of participants and are therefore not representative of the general population. Furthermore, the majority of these studies were conducted on pregnant women receiving ANC services in health facilities, which may have inflated their findings. As a result, the current study used a two-stage multilevel mixed-effects logistic regression model with Mini-EDHS 2019 data to identify individual and community-level predictors of iron to folic acid supplementation during pregnancy.

## Methods

### Study area and data source

The data were retrieved from the Ethiopia Mini Demographic and Health Survey (DHS), 2019 program’s official database website.^1^ Ethiopia is a Federal Democratic Republic with 11 geographical regions where the two are city administrations. Regional states are divided into zones, and zones are subdivided into woredas, and woredas into kebeles, the lowest administrative units. The 2019 mini-DHS were conducted in all the geographic areas of the country from March 21, 2019, to June 28, 2019. In terms of its population size, Ethiopia is the second most populous nation in Africa with more than 112 million people (56,010, 000 Females and 56, 069, 000 Males) in 2019. Women of reproductive ages (15–49 Years) represent 21% of the population in the country (MDHS, 2019).

### Study participants

The Mini Ethiopian Demographic and Health Survey (MEHDS) was conducted to reproductive-age women. This study targeted women between the ages of 15 and 49 years who had their last child within the 5 years preceding the survey in Ethiopia, EMDHS 2019. A weighted 2,356 women who had received IFA supplements and were asked how many days they consumed IFA tablets/ syrup during their last pregnancy that occurred 5 years prior to the mini national survey were included in the sample, and the dataset we used was the maternal dataset.

### Study variable

Dependent variable: The outcome variable of this study was IFA supplementation adherence level.

IFA adhered: Those mothers who took the supplement of IFA for the recommended periods which is 90 days or more.

Not adhered: mothers who took the IF supplement for less than the recommended periods (less than 90 days).

Independent variables: All the independent variables were classified into individual-level and community-level variables.

Individual-level variables: Maternal age, religion, wealth index, women’s education level, parity, ANC visits, birth interval, number of children ever born marital status, head of the household and number of ANC visits.

Community-level variables: The two non-aggregate community-level factors were place of residence and region. Community women illiteracy, poverty, and no ANC visit were variables derived from aggregating individual-level variables. Based on the distribution of the average values calculated for each cluster, each aggregate variable was classified as “high” or “low.” The median value was used for aggregate variables, and the median value was used as a cut off point for categorization. As a result, these variables were measured in the following manner.

### Community women’s illiteracy level

Low: The proportion of illiterate below the median of illiteracy level.

High: The proportion of illiterate higher than the median illiteracy level.

### Proportion of no ANC at the community

Low: The proportion of no ANC visit in the clusters below the median level.

High: The proportion of no ANC visit in the clusters median and above level.

### Community poverty level

Low: The proportion of poor below the median of poor wealth index.

High: The proportion of poor above the median of poor wealth index.

### Data analysis

The analysis was carried out using Stata 17.0. The data were weighted using a primary sampling unit to restore representativeness and obtain a reliable estimate. The proportions and frequencies were estimated weighted for each variable. This was based on a thorough explanation of the mini-EDHS sample weighting procedure (23). Continuous variables were categorized and re-categorize using information obtained from various works of literature. The variables were described using descriptive statistics.

Due to the hierarchical nature of the 2019 EDHS data, a multilevel mixed effect binary logistic regression analysis was fitted in this study to estimate the effects of individual and community-level determinants of IFA supplementation non-adherence.

We have employed a complex multilevel data analysis technique (melogit [pweight = wt] || v001:) to adopt a comprehensive approach in examining intricate survey data through a multilevel logistic regression model. By utilizing this method, we effectively address the complexities of the survey design and ensure the provision of accurate statistical inferences.

To select potential variables for multivariable analysis, bivariable multilevel binary logistic regression analysis was performed on each independent individual and community level variable. Finally, multivariable multilevel analysis was performed, and variables that showed a significant association at *p* value less than 0.05 with adjusted odds ratio and 95% CI were reported as statistically significant factors for IFA supplementation non-adherence in Ethiopia.

### Model building and selecting the best-fitted model

To identify the potential factors associated with non-adherence to iron folic acid supplementation, a multilevel mixed effect binary logistic regression was fitted. The null model (Model I) was fitted first. The variance of the random effect in this model was 0.71, indicating that there is significant variation across clusters. There was also Intra-Custer Correlation (ICC = 17.72%), indicating that 17.72% variability in non-adherence levels was due to clusters. Model II was fitted after bivariable analysis by adding individual level factors to the null model. Model III was then fitted by incorporating the community level variables into the null model. Finally, model IV was fitted by combining individual level and community level factors. The final model (Model IV) was appropriate to identify the individual and community level factors of non-adherence to iron folic acid supplementation in Ethiopia after checking the model fitness level using different post estimation methods (AIC, BIC, and loglikelihood) (Table 1). Variable Inflation Factors (VIF) and tolerance level were used to check for multicollinearity among independent variables, and there was no collinearity across variables.

**Table 1:** Variability at community-level and model comparison for iron folic acid supplementation adherence among women age 15–49 with a child born in the 5 years preceding the survey in Ethiopia, EMDHS 2019.

Parameters	Null model	Model II	Model III	Model IV
ICC	17.72%			
AIC	2198.684	2200.598	1547.79	1535.278
BIC	2210.171	2217.829	1613.219	1649.778
Log likelihood	−1097.342	−1097.299	−761.8949	−746.639

## Results

### Socio-demographic characteristics of women

The data were first weighted. The analysis included 2,356 women who were given/brought for supplementation during pregnancy out of a total of 3,927 women aged 15–49 with the last child born in the 5 years preceding the survey. Of the total respondents, 1,256 (53.33%) were between the age group of 25–34 years, nearly (42%) had no formal education, and the majority (94.1%) were currently in union. The majority of the mothers were Orthodox Christian (44.81%) followers, 46.96% of respondents were from the rich households, and 86.95% had 6 or more children ever born. From the total number of participants, 69.61% were from rural areas, and 867 (36.81%) were from Tigray and Amhara regions. Almost 1,100 women (46.69%) come from wealthy families, while 769 (32.66%) come from low-income families. Almost 86.95% of the households were headed by a man, and the majority of women (83.74%) had a birth interval of 2 years or more between the births of the index child (Table 2).

**Table 2:** Socio-demographic characteristics of Ethiopian women aged 15–49 with a child born in the 5 years preceding the survey, EMDHS 2019.

Variables	Category	Frequency	Percentage
Maternal age	15–24 years	625.5	26.55
	25–34 years	1,256.2	53.33
	35–39 years	308.9	13.11
	40–49 years	164.9	7.00
Maternal educational level	No education	986.9	41.89
	Primary	956.99	40.63
	Secondary and higher	411.71	17.48
Religion	Orthodox	1,055.5	44.81
	Muslim	700.2	29.73
	Protestant	571.5	24.26
	Others	28.35	1.20
Residence	Urban	715.77	30.39
	Rural	1,639.78	69.61
Region	Tigray_Amhara	866.99	36.81
	Oromia	832.5	35.34
	SNNPR	434.45	18.44
	Most urbans	114.84	4.88
	Eastern pastoralist	66.86	1.69
Marital status	Currently in union	2,216.3	94.1
	Currently not in union	139.3	5.9
Household wealth index	Poor	769.38	32.66
	Medium	486.27	20.64
	Rich	1,099.90	46.69
Community women education(illiterate)	Low	1,272.65	54.03
	High	1,082.91	45.97
Community poverty	Low	1,393.39	59.15
	High	962.16	40.85
Birth interval	Less than 2 years	289.67	16.26
	Two and more years	1,491.97	83.74
Number of children ever born	Less than 6	1,889.75	80.22
	6 and above	465.81	19.78
Head of the household	Male headed	2,048.07	86.95
	Female headed	307.49	13.05

In terms of community educational level, 1,273 (54.03%) of women in the community had low literacy level, and 1,393.39 (59.15%) of the community had low poverty level (Table 2).

### Women’s characteristics related to maternal health service utilization

In this survey, 271 (11.49%) women were confirmed pregnant at the time of the survey, and 56.26 percent began ANC during the second trimester. The majority of the women (56.20%) was multiparous and had four or more ANC visits during their pregnancy (57.60%). One thousand three hundred thirty-seven (56.76%) of the women in the community have low proportion of not having ANC visits (Table 3).

**Table 3:** The characteristics of maternal health service utilization of women aged 15–49 who had a child in the 5 years preceding the MEDHS 2019.

Variables	Category	Frequency	Percentage
Parity	uni para	565.94	24.03
	Multi para	1,323.81	56.20
	Grand multipara	465.81	19.78
Currently pregnancy	no or unsure	2,084.99	88.51
	Yes	270.56	11.49
Timing of first ANC visit	First trimester	853.33	36.23
	Second trimester	1,325.20	56.26
	Third trimester	177.02	7.52
Number of ANC visit during pregnancy	No ANC visit	106.11	4.50
	1–3 ANC visit	892.73	37.90
	4 and above ANC visit	1,356.72	57.60
Proportion of no ANC in the community	Low	1,337.04	56.76
	High	1,018.52	43.24

### The magnitude of adherence level to iron folic acid supplementation of pregnant women in Ethiopia

**Figure 1:**
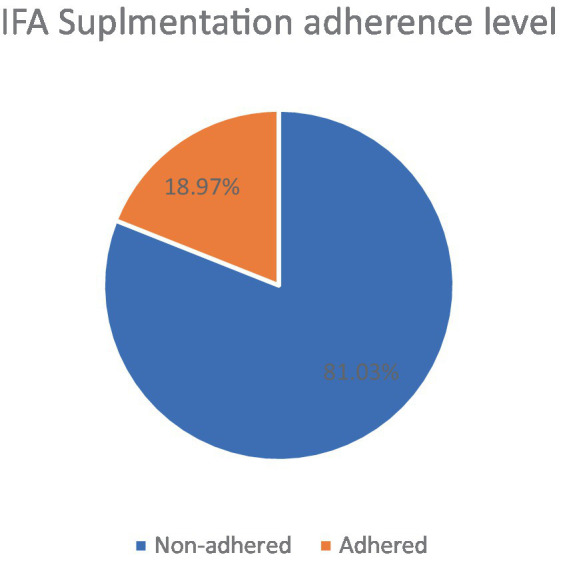
Iron folic acid supplementation adherence level among women age 15–49 with the last child born in the 5 years preceding the survey in Ethiopia, MEDHS 2019.

Iron folic acid supplements were given to 2,356 women (59.99%). Among those, 1,909 (81.03% (95% CI: 79.39, 82.56)) of women did not follow WHO recommendations for iron folic acid (IFA) supplementation, while 18.97% of women in Ethiopia adhered to iron folic supplementation in Ethiopia (Figure 1).

### Factors associated with non-adherence to iron folic acid supplementation

To identify the potential factors associated with non-adherence to iron folic acid supplementation, a multilevel mixed effect binary logistic regression was fitted. The final model (Model IV) was appropriate to identify the individual and community level factors of non-adherence to iron folic acid supplementation in Ethiopia after checking the model fitness level using different post estimation methods (AIC and loglikelihood) (Table 1). According to the multilevel multivariable analysis results Birth interval, the number of children ever born, timing of First ANC visit, region, and proportion of no ANC in the community were statistically significant factors of non-adherence to iron folic acid supplementation in Ethiopia (Table 4).

**Table 4:** Individual and community level factors associated with iron folic acid supplementation adherence among women age 15–49 with the last child born in the 5 years preceding the survey in Ethiopia, MEDHS 2019.

Variables		IFA supplantation adherence level		COR at 95% CI	AOR at 95% CI
		Non adhered	Adhered		
Age of the women	15–24 years	485.36	140.14	1	1
	25–34 years	1,023.09	233.10	0.70(0.46, 1.07)	1.14(0.52, 2.48)
	35–49 years	400.14	73.74	0.57(0.34, 0.95)*	1.44(0.60, 3.45)
Religion	Orthodox	865.26	190.26	1	1
	Muslim	542.72	157.49	1.40(0.90, 2.14)	1.70(0.91, 3.19)
	Protestant	476.11	95.36	0.95(0.52, 1.74)	1.68(0.73, 3.82)
	Others	24.49	3.85	0.53(0.054,5.25)	1.22(0.08, 17.48)
Birth interval	Less than 2 years	217.57	72.10	1.71(0.93, 3.14)*	2.03(1.12,3.66)*
	Two and more years	1,249.69	242.28	1	1
Number of children ever born	Less than 6	1,504.81	384.94	1.96(1.17, 3.29)*	1.99(1.09, 3.64)*
	6 and above	403.79	62.03	1	1
Timing of first ANC visit	First trimester	636.41	216.93	3.80(1.47, 9.80)*	2.74(1.03, 7.30)*
	Second trimester	1,112.37	212.83	2.06(0.79, 5.344)	1.61(0.59, 4.39)
	Third trimester	159.81	17.21	1	1
Household head	Male	1,648.90	399.17	1	1
	Female	259.69	47.80	0.64(0.44, 0.92)*	0.64(0.36, 1.15)
Region	Tigray_ Amhara	705.27	161.73	1	1
	Oromia	633.66	198.83	1.5(0.96, 2.35)	1.23(0.61, 2.49)
	SNNPR	399.66	34.79	0.37(0.21, 0.67)*	0.24(0.10, 0.61)*
	Most urbans	81.49	33.35	2.02(1.38, 2.95)*	1.44(0.73, 2.84)
	Eastern pastoralist	57.87	8.99	0.72(0.38, 1.35)	0.49(0.19,1.29)
	Western semi-pastoralist	30.65	9.27	1.42(0.87, 2.30)	1.53(0.69,3.39)
Residence	Urban	567.28	148.49	1	1
	Rural	1,341.31	1,639.78	0.73(0.50, 1.06)	1.16(0.61, 2.20)
Community illiteracy level	Low	1,008.38	264.26	1	1
	High	900.21	182.70	0.91(0.64, 1.30)	0.99(0.51, 1.90)
Community poverty	Low	1,103.67	289.72	1	1
	High	804.92	157.25	0.74(0.52,1.05)	0.68(0.39, 1.18)
Proportion of no ANC in the community	Low	1,076.53	260.51	1	1
	High	832.06	186.46	0.95(0.66, 1.36)	1.77(1.08, 2.88)*

*Indicates variable which have association with outcome variable.

The odds of non-adherence to iron folic acid supplementation of pregnant women having birth interval less than 2 years were 2times higher than having two and above years birth interval [AOR: 2.03; 95%CI: 1.12,3.66; at *p* < 0.05]. The odds of non-adherence to iron to folic acid supplementation of pregnant women ever born less than 6 children were also 1.99 times higher than whoever born 6 and more children [AOR: 1.99; 95% CI: 1.09, 3.64 at *p* < 0.05]. The timing of first ANC visit was also significant factor for non-adherence. Those mothers who started there ANC visit during first trimester were 2.74 times more likely become non-adherence to iron folic acid supplementation when compared to who started in third trimester [AOR: 2.74; 95% CI: 1.03, 7.30 at *p* < 0.05].

On the other hand, mothers from SNNPR were decreases the non-adherence to IFA supplementation by 76% when compared to Tigray Amhara region [AOR = 0.24; 95%CI: 0.10, 0.61 at *p* < 0.01]. The odds of non-adherence to iron folic acid supplementation also were 1.77 times higher among higher Proportion of no ANC in the community than counter parts [AOR = 1.77; 95%CI: 1.08, 2.88 at *p* < 0.05] (Table 4).

### Variability at community level and model comparison to select the appropriate model

Variability at community-level and Model comparison for iron folic acid supplementation adherence among women age 15–49 with a child born in the 5 years preceding the survey in Ethiopia, EMDHS 2019 as shown in Table 4.

## Discussion

Iron folic acid supplementation is critical in the prevention and treatment of iron deficiency anemia, especially in pregnant women whose iron requirements increase due to fetal and maternal needs (24). Thus, the purpose of this study was to assess the level of non- adherence to iron folic acid supplementation during pregnancy and its associated factors among Ethiopian women aged 15–49 years who had the last child in the previous 5 years using MEDHS 2019. The overall magnitude of non-adherence to iron folic acid supplementation was high in Ethiopia. Birth interval, number of children ever born, and time of ANC visit were individual level factors that were significantly associated with non-adherence to iron folic acid supplementation during pregnancy in Ethiopia, whereas region and proportion of community no ANC visit were community level factors.

In Ethiopia, the overall magnitude of non-adherence to iron folic acid supplementation during perinatal periods was 81.03% among women aged 15–49 years. This finding is higher than the meta-analysis study conducted in Ethiopia (53.85, 58.62%) (13, 14), different single study reports in Ethiopia (24–27), 22 African countries DHS data (71.3%) (11), DHS data report of Haiti (68%) and Malawi (65%) (28), meta-analysis in SSA (60.8%) (29) and the study done in Kenya (30). The magnitude of non-adherence in this study was also slightly lower than the magnitude of non-adherence to iron folic acid supplementation reported in Ethiopia using EDHS 2016 (86.6%) (31, 32) and the magnitude of non-adherence in northern Ethiopia (89.5%) (33). Furthermore, according to the EDHS 2016, 95% of pregnant women took iron folic acid for less than 90 days. The difference could be due to the study design, as the current study used a large-scale survey, whereas the others used meta-analysis and a single survey in a limited area. Another explanation for the variation could be recall bias for EDHS data, which increases non-adherence while most individual studies are conducted at the institution level during the time of ANC follow up. Furthermore, this finding was lower than the EDHS, 2016 different reports, and this is due to various interventions made between 2016 and 2019. Another reason for the variation could be due to study periods and location.

In this study, pregnant women with birth intervals of less than 2 years had twice the odds of not taking IFA supplements as compared with birth intervals of 2 years or more. This finding is consistent with the findings of a study on Folic Acid Supplementation and Interpregnancy Interval in Norway (34) and a study in Tanzania (35). The shorter birth intervals were primarily due to a lower proportion of planned pregnancies (34). Because of the unplanned pregnancy, the iron folic acid supplementation is not taken as recommended.

Mothers who have had fewer than six children are more likely to have poor adherence for IFA supplementation than mothers who have had six or more children.

Mothers with less than six children are more prone to non-adherence to iron and folic acid (IFA) supplementation due to several justifiable reasons. Firstly, mothers with fewer children often have a relatively younger age, which can be associated with a lack of experience and knowledge regarding the importance of IFA supplementation during pregnancy. They may be less aware of the potential risks and benefits of IFA supplementation, leading to a higher likelihood of non-adherence.

Secondly, mothers with six or more children tend to have a higher level of experience and familiarity with pregnancy and maternal health practices. They have likely received more exposure to healthcare information and interventions, including IFA supplementation, throughout their previous pregnancies. This accumulated experience and knowledge may contribute to a higher level of adherence among this group. Therefore, the higher level of adherence among mothers with six or more children can be attributed to a combination of factors, including increased awareness, experience, knowledge, and support. Timing of the first ANC visit was one of the significant factors for non-adherence to IFA supplementation during pregnancy. This study found that mothers who started their ANC in the first trimester were 2.7 times more likely to be non-compliant than mothers who started in the third trimester. This finding contradicts the findings of a study conducted in Gondar and Debre Tabor hospitals, which found that beginning the first ANC visit increases IFA supplementation adherence (25, 27) and Ethiopian ANC strategy which demonstrated that initiating the first ANC visit actually increased adherence to IFA supplementation. During pregnancy, it is best to begin iron and folic acid supplementation as soon as possible. Most Ethiopian women do not begin iron folic acid supplementation during the first trimester of pregnancy because the majority of them discover their pregnancy after the first trimester and begin ANC during the second half of pregnancy (36).

This delay in starting IFA supplementation is often attributed to late pregnancy detection, as many women only discover their pregnancy after the first trimester and subsequently initiate ANC visits.

Interestingly, when women do begin ANC visits in the first trimester, they may also commence IFA supplementation during this time. However, this early initiation of IFA supplementation can lead to physiological disturbances, particularly gastrointestinal discomfort, which commonly occurs during the first trimester. Consequently, some women may discontinue taking IFA due to these adverse effects. This initial negative reaction to IFA tablets during the first trimester can have a lasting impact on adherence to IFA supplementation in subsequent months of pregnancy, ultimately resulting in non-compliance with the World Health Organization’s (WHO) recommendations.

It is recommended to start IFA supplementation early in pregnancy, the timing of the first ANC visit plays a significant role in adherence to IFA supplementation. The contradictory findings between studies emphasize the need for further research and tailored interventions to ensure optimal adherence to IFA supplementation throughout pregnancy, taking into account the specific context and challenges faced by pregnant women.

We found that IFA supplement non-adherence is associated with clusters with a higher proportion of no ANC in the community. Non-adherence to iron folic acid supplementation was also 1.77 times higher in communities with a higher proportion of no ANC than in others. This finding is consistent with Ethiopian studies (32, 37), Haiti and Malawi (28). This could be because an ANC visit is the entry point for utilizing maternal health care services such as IFA supplementation. This fosters in the community positive compliance behavior in the use of IFA supplements.

The findings also revealed that there is variation in IFA supplement adherence across regions, which is treated as a community level variable. Women in SNNPR had higher levels of adherence than women in northern Ethiopia (Amhara-Tigray). Mothers residing in the SNNPR region exhibited a significantly lower rate of non-adherence to IFA supplementation, with a reduction of 76% compared to mothers residing in the Tigray Amhara region. This finding contradicts previous research findings in Ethiopia (13). The observed variation in non-adherence to IFA supplementation between the SNNPR and Tigray Amhara regions can be attributed to intensified intervention efforts by governmental and non-governmental organizations. These interventions were implemented in response to previous studies reporting inadequate adherence and supplement utilization in the SNNPR region. It is possible that this discrepancy reflects the regional and federal government’s commitment to enhancing intervention strategies, particularly in maternal services, with a specific focus on improving adherence to IFA supplementation. In this study; we tried to investigate individual and community-level factors associated with non-adherence to IFA supplementation in Ethiopia. As a result, it is more representative, and policymakers and other stakeholders can use the study’s findings to plan and implement effective strategies and interventions. This study, however, has some limitations. Because the outcome variable was assessed based on the maternal report within the 5 years preceding the survey, the study was subject to recall bias. Furthermore, some of the data was incomplete and difficult to analyze.

## Conclusion and recommendations

In Ethiopia, nearly four out of every five pregnant women did not receive iron folic acid supplementation for the recommended periods. Birth intervals of less than 2 years, women having fewer than six children, and beginning ANC visits during the first trimester were individual level factors that increase the non-adherence to iron folic acid supplementations. Non-adherence to iron folic acid supplementation in Ethiopia was also influenced by region and community level on ANC service. Thus, the following recommendations are important.

Promote birth spacing: Encourage women to have a birth interval of at least 2 years between pregnancies. This can be done through family planning programs and education on the importance of birth spacing for maternal and child health.

Enhance maternal health services: Focus on women who have had fewer than six children, as they were found to have a higher likelihood of non-adherence. Strengthen antenatal care (ANC) services by ensuring early initiation of ANC visits during the first trimester. This can be achieved through awareness campaigns and improving access to quality ANC services.

Regional interventions: Consider regional variations in non-adherence rates and tailor interventions accordingly. Identify regions with higher non-adherence rates and implement targeted strategies to address the specific challenges and barriers faced in those areas.

## Data availability statement

The datasets presented in this study can be found in online repositories. The names of the repository/repositories and accession number(s) can be found in the article/supplementary material.

## Ethics statement

Authorization to download and use the Mini EHDS, 2019 dataset for this study was obtained from the DHS Program/ICF International following the request made by the Investigators. The data obtained for this study has no personal level identifiers and hence there is no way those respondents’ privacy and confidentiality of the information be broken.

## Author contributions

HT: Data curation, Software, Validation, Writing – original draft, Writing – review & editing. WW: Conceptualization, Formal analysis, Methodology, Software, Writing – original draft, Writing – review & editing. FF: Data curation, Methodology, Software, Validation, Visualization, Writing – original draft, Writing – review & editing. GM: Methodology, Software, Writing – review & editing. KT: Methodology, Software, Writing – review & editing. TA: Methodology, Validation, Writing – review & editing.

## Footnotes

^1^https://dhsprogram.com/data/available-datasets.cfm
